# Barriers to Follow-Up for Abnormal Papanicolaou Smears among Female Sex Workers in Lima, Peru

**DOI:** 10.1371/journal.pone.0169327

**Published:** 2017-01-06

**Authors:** Devora Aharon, Martha Calderon, Vicky Solari, Patricia Alarcon, Joseph Zunt

**Affiliations:** 1 Icahn School of Medicine at Mount Sinai, New York, NY, United States; 2 Centro de Salud Alberto Barton del Callao, Callao, Lima, Peru; 3 Pontificia Universidad Catolica del Peru, Callao, Lima, Peru; 4 University of Washington, Seattle, WA, United States; Universidade Estadual de Maringa, BRAZIL

## Abstract

**Background:**

Cervical cancer is the most prevalent cancer among Peruvian women. Female sex workers (FSW) in Peru are at elevated risk for HPV infection, and receive annual Papanicolaou screening. The objective of this study was to identify barriers to follow-up for abnormal Pap smears among FSW in Peru.

**Methods:**

97 FSW attending the Alberto Barton Health Center in Lima were surveyed regarding their STI screening history. 17 women with a history of an abnormal Pap smear were interviewed about their experiences regarding follow-up care.

**Results:**

Of the 27 HPV-positive women, only 8 (30%) received follow-up treatment. Of the 19 women who did not receive follow-up, 7 (37%) had not been informed of their abnormal result. Qualitative interviews revealed that the major barrier to follow-up was lack of knowledge about HPV and potential health consequences of an abnormal Pap smear.

**Conclusion:**

HPV infection is highly prevalent in Peruvian FSW, yet only 30% of FSW with abnormal Pap smears receive follow-up care. The predominant barriers to follow-up were lack of standardization in recording and communicating results and insufficient FSW knowledge regarding health consequences of HPV infection. Standardization of record-keeping and distribution of educational pamphlets have been implemented to improve follow-up for HPV.

## Introduction

Cervical cancer is increasingly preventable with the advent of screening, treatment, and vaccination against human papillomavirus (HPV), a sexually transmitted infection responsible for 99 percent of cervical cancer cases worldwide [1]. Although cervical cancer has decreased to the tenth most prevalent type of cancer among women in high-income countries, it remains the second leading type of cancer among women in low- and middle-income countries (LMICs) [2] and accounts for 87% of cervical cancer deaths in LMICs [3]. Although screening programs for HPV have been present in Latin America for over 30 years, Latin American countries have not witnessed the decrease in mortality from cervical cancer seen in high-income countries [4]. A Colombian study found that increased screening coverage did not correlate with reductions in cervical cancer mortality; however, a reduction in mortality was seen in areas where a higher proportion of women sought medical advice related to an abnormal Pap smear [5]. Effective follow-up for abnormal screening tests in underserved areas may therefore be the key to closing the gap in morbidity and mortality from this largely preventable disease.

In Peru, cervical cancer is the leading cause of cancer among women and the second highest cause of death from cancer in women [6]. Despite availability of screening procedures, cervical cancer deaths in Peru increased between 2000 and 2010 [7]. While this increase may be partially attributable to improved detection and record keeping, it suggests that the presence of screening programs alone is insufficient to prevent cervical cancer deaths. Follow-up care for abnormal Pap smears in Peru may be difficult to access, especially as the current system for providing indigent care covers screening but not treatment [8].

Female sex workers (FSW) are at increased risk of acquiring high-risk type HPV infection compared to the general population [9,10], and when proper screening and treatment protocols are not present, are at additional risk for developing cervical cancer. In Lima, Peru, where sex work is permitted, registered FSW receive regular screening for sexually transmitted infections (STI) at designated health clinics such as the Centro de Salud “Alberto Barton” del Callao (Alberto Barton Health Center of Callao). Studies have demonstrated a prevalence of cervical HPV among FSW in Lima of 50.6–66.8%, including high-risk types in 35.6–42.0%, thus highlighting the need for an intervention to ensure that women with high-risk HPV infection receive appropriate treatment to prevent progression to cervical cancer [9, 10].

All sex workers registered to work in Callao must attend monthly screening visits at the Barton Clinic and adhere to strict screening protocols. The Barton Clinic screens an estimated 300 FSW for HPV and cervical dysplasia each month. However, the clinic lacks resources to provide treatment for abnormal test results, and must refer women with positive findings to outside hospitals for care. Given that screening and follow-up care are provided at different locations, there is a chance that women will not follow through with treatment. Additionally, barriers to follow-up such as lack of access to resources, stigmatization, and psychosocial factors have received little attention. We assessed barriers to follow-up of abnormal Pap smears among female sex workers in Lima, Peru as a first step to improving access to appropriate treatment of abnormal Pap smear and high risk HPV infection.

## Methods

All female sex workers attending Barton for monthly STI screening exams were invited to participate. A questionnaire asked about the patient’s history of receiving results for sexually transmitted infections screened for at Barton, including HPV, cervicitis, vaginosis, syphilis, candidiasis, trichomoniasis, and HIV. Patients were also asked about whether they received follow-up care for the respective STI for which they screened positive, and reasons why they received follow-up or perceived barriers to not receiving follow-up. Follow-up care was defined as any diagnostic or therapeutic procedure related to the initial screening test result.

Women who reported having tested positive for HPV or abnormal cytology in the past were invited to participate in an in-depth interview. Review of medical records was also conducted for all study participants in order to identify cases of positive cervical cancer screening, and these women were invited to participate in in-depth interviews as well. Interviews addressed the participant’s experience of having an abnormal Pap smear and why the participant did or did not receive follow-up care. Topics addressed during interviews included understanding of the disease process, understanding of how to access care, economic barriers, potential inconveniences, relationships with the health care system and personnel, influence of family and friends, and emotional factors including stigma and fear. Interviews were conducted by a Peruvian psychologist who was not a member of the Barton staff. An Interview Guide provided a framework of questions in order to cover similar information in each interview.

Questionnaires were analyzed using STATA, and in-depth interviews were analyzed using NVivo software (QSR International, Victoria, Australia).

The study took place from March 2014 to July 2014. The study was approved by the Institutional Review Board of the University of Washington and the Research Ethics Committee of the Directorate of Health (DIRESA) Callao, Peru. Informed consent was obtained orally to protect the identities of study participants. Oral consent was approved by both ethics review committees.

## Results

Ninety-seven women completed questionnaires. Participant demographics are outlined in Table 1. Average age of participants was 34 years, average level of education was secondary school, average years of work as a sex worker was 5.6 years, average yearly income was $30,000, and average age at first sex was 16 years.

**Table 1:** Participant demographics.

Parameter	N (%)	Average	Range
**Age**		34 years	19–72 years
18–30	44 (46%)		
30–40	23 (24%)		
40–50	20 (21%)		
>50	8 (8%)		
**Education level**		Secondary school	Primary school–Professional degree
Professional degree	3 (3%)		
University degree	12 (12%)		
Secondary school	46 (48%)		
Primary school	21 (22%)		
Some primary school	13 (14%)		
**Years of work as FSW**		5.6 years	1 month-37 years
0–1	25 (26%)		
2–5	39 (41%)		
6–10	16 (16%)		
>10	15 (15%)		
**Yearly income (Peruvian Soles)**		$30,000	$6,500-$70,000
<1000	2 (2%)		
1001–2000	28 (29%)		
2001–5000	41 (43%)		
5001–10000	22 (23%)		
>10000	1 (1%)		
**Age at first sex**		16 years	3–24 years
0–10	2 (2%)		
11–15	44 (46%)		
16–20	41 (43%)		
>20	8 (8%)		

### Quantitative analysis

Of the 97 women who completed questionnaires, 27 were identified as having had abnormal cervical screening by Papanicolaou smear. Of the 27 women with a history of abnormal Pap smear, only 8 (30%) had received follow-up treatment. Of the 19 women who did not receive follow-up care, 7 (37%) had not been informed of the result of their abnormal screening test. In contrast, patient-reported follow-up rates for other positive STI screening tests approached 100% (Table 2).

**Table 2:** Patient-reported prevalence and follow-up for STI.

Sexually Transmitted Infection	Number of Patients (%)	Follow-up care for abnormal result
Cervicitis	56 (58%)	98%
Candida	43 (44%)	100%
Bacterial Vaginosis	43 (44%)	98%
HPV/Abnormal Pap	27 (27%)	30%
Syphilis	12 (12%)	100%
Genital Herpes	5 (5%)	80%
Trichomoniasis	5 (5%)	100%
HIV	1 (1%)	100%

### Qualitative analysis

Seventeen women participated in qualitative interviews: 5 women who had received follow-up care, and 12 who had not. Of the 12 women who had not received care, 5 had never been informed of the result of their cervical screening test, and 7 were aware of the result but had not received care. After all women were informed of their diagnosis, an additional 3 women received follow-up care prior to their interview. Therefore, interviews were conducted with 8 women who received follow-up care (follow-up group, FUG) and 9 women who did not receive follow-up care (no-follow-up group, NFUG).

#### Factors associated with lack of follow-up

A major barrier to receiving follow-up care was not having been informed of abnormal test results. The clinic lacked structured procedures for recording when the cervical screening test had been performed, the result of the test, and whether results had been transmitted to the patient. One participant reported having asked for her test result three months after her screening test had been performed. The result was not located in her chart, and clinic personnel searched in the master Pap screen registry to find her result. Although the result had been entered into the registry, the patient would not have received the positive result if she had not requested it.

Women who did not receive follow-up care tended to have less knowledge regarding the association between HPV infection and cervical cancer. They were also less likely to know that the Pap test is a screening test for cervical cancer, and that a positive screening test can be monitored and/or treated to prevent the development of cervical cancer. Only 3 of the 9 NFUG women identified cancer as a potential consequence of a positive screening exam, whereas all 8 women in the FUG reported cancer as a potential consequence.

Knowing someone who had had a negative experience with follow-up for an abnormal Pap smear influenced three women not to receive care. One woman in the NFUG reported, “A friend from work…showed me pictures of what they cauterized in the uterus but they were horrible, horrible…and that it hurt, I couldn’t see any more.”

Being a migrant worker was also associated with lack of follow-up care. A number of women had come to Peru from nearby countries including Ecuador and Colombia to work, and women often traveled to and from their home country for months at a time. Three women attributed their lack of follow-up care to being outside of Peru.

#### Factors associated with successful follow-up

Women who received follow-up care were more likely to have heard of HPV and its relationship to cervical cancer. 8 out of 8 women in the FUG verbalized that there was an association between their positive screening result and cancer risk, while only 6 out of 9 women in the NFUG verbalized this association. The 8 women in the FUG also had more in-depth knowledge regarding HPV and cervical cancer. They were more likely to know that the Pap test screens for cervical cancer and that appropriate follow-up care can help prevent development of cancer. In response to the question of why she chose to receive follow-up care, one woman said, “So that I can be cured quickly of this infection…I was afraid, the doctor told me that there are three classes, 1, 2, and 3, and 2 is precancer, but 1 is only inflammation.” This participant feared escalation of the disease to cancer, but also understood that prevention was possible, and this information motivated her to seek care. However, misinformation was present in the FUG as well. One woman reported that she had been told that she had cancer, though her diagnosis was CIN2 (cervical dysplasia). She said, “I heard the word ‘cancer’ and I had to sit, and I forgot everything, I completely forgot what I was going to do and just stayed there…I only heard cancer, cancer, you are going to die…” While this misinformation motivated her to receive timely treatment, this created unnecessary fear and anxiety as well as conflict within her family.

Presence of social support was also associated with obtaining follow-up care. 5 FUG women had spoken to a family member, partner, or friend about their diagnosis, their emotional response, and the decision to receive care, compared to 2 NFUG women. These FUG women reported encouragement from loved ones to pursue further diagnostic workup and/or treatment, and for some, support from a loved one was crucial to their decision to seek care: “When they gave me the result I didn’t want to know anything about it, I didn’t want to come back, but [my partner] told me to come to be really sure…about what I had.”

Having an acquaintance who had died from cervical cancer influenced some participants to receive follow-up care. Two FUG women reported knowing someone who died from cervical cancer, while no one in the NFUG group reported this. This experience led to increased familiarity with the screening, follow-up procedures, and potential consequences related to HPV and cervical cancer. According to one woman, a family history of cancer was influential in her decision to seek screening and follow-up. “My grandmother died of cervical cancer…and they said it was because she hadn’t come in earlier. My mother also did her Papanicolaou and mammography.”

#### Factors not associated with obtaining follow-up care

A number of factors were present equally in both groups. All women reported a high level of satisfaction with their providers and the medical system. Women in both groups attributed their decision to pursue or not pursue follow-up care to fear: 6 FUG women and 6 NFUG women reported feeling fearful of the diagnosis and its consequences. Although 6 FUG women and 4 NFUG women felt ashamed of their diagnosis, none of the women attributed their decision to their level of shame.

The length of delay between the time the test was performed and the time the diagnosis was received was not associated with obtaining follow-up care. One woman who had not received follow-up had had a positive screen 5 years prior to the study period, but scheduled a colposcopy immediately after she received her result. The majority of women had been screened between 1 and 5 months prior to the study period, with an even distribution of time periods between groups who had and had not received follow-up.

Economic factors were not associated with obtaining follow-up care. Three FUG women reported that cost was a barrier to receiving care, while two NFUG women reported that cost of treatment was a barrier. One FUG woman reported that her main concerns were the fear of developing a malignancy, the cost of treatment and the time necessary to obtain treatment.

Despite needing to independently locate a follow-up facility, none of the women surveyed reported this as a barrier to follow-up care. Finally, needing to miss work days to be treated and recover was a challenge present equally in both groups.

## Discussion

Our study highlights that a cervical cancer screening program alone is not sufficient to ensure women receive proper treatment. Follow-up of abnormal Pap smear was only 30% among female sex workers in Peru. Barriers to follow-up included not receiving Pap smear or HPV test results, lack of record-keeping at the clinic to track which women had received results, lack of understanding of the test results–namely, that HPV is a cause of cancer–and lack of knowledge regarding cervical cancer. While factors such as economic cost, missed days working, and shame were challenges for many FSW with abnormal Pap smears, these factors did not differ between women who did or did not seek follow-up care.

Consistent with our findings, studies of other underserved populations have reported that inadequate communication was a major barrier to follow-up. In Jamaica, good rapport with clinic personnel positively predicted adherence to follow-up for abnormal cytology [11]. Among Latina women in the U.S., major barriers included inadequate communication about the diagnosis and follow-up appointments or procedures, as well as anxiety/fear, interference with work or childcare, and pain [12]. In Nicaragua, major reasons for not attending follow-up for an abnormal Pap smear were postponement by the center, pregnancy, financial constraints, or a lack of electricity or instruments [13]. Other factors that have been attributed to lack of follow-up care for abnormal Pap smears include: lower socioeconomic status, poor social support, fear, inconvenience, and higher dysplasia severity [14,15].

Our study also found that follow-up was more common in those women who received and understood their test results, as well as the potential consequences upon their health. Motivation to seek care was also associated with increased level of knowledge and understanding of the test results and disease process. Regardless of whether they had or had not received their results, nearly all women recommended that the clinic provide more information to patients regarding HPV infection and cervical cancer, as well as additional counseling services.

The disparity between rates of follow-up for HPV or for other STI is likely associated with the delays in obtaining Pap smear and HPV test results. Whereas rapid tests provide results for many STI the same day as the clinic visit, allowing treatment to be prescribed the same day, cytology examinations are sent to an outside laboratory, with the results provided at a subsequent visit. Additionally, follow-up care for abnormal cytology is via referral by the Barton Clinic to a local hospital.

One limitation of this study is that interviews were conducted only at the Barton Clinic. This location was chosen because the clinic is a safe and familiar environment for the FSW who have received care at the clinic for months or years. While non-clinic personnel conducted interviews, the location may have limited the validity of the reported satisfaction with providers and the medical system or generalizability to other female sex worker populations in other countries.

Following this study, two interventions were implemented at the Barton Clinic in an attempt to increase follow-up rates. Standardized forms for recording Pap smear exam dates, results, and follow-up care were created and added to medical charts. Additionally, a pamphlet regarding Pap smears, HPV, and cervical cancer was created to distribute to FSW at Barton at the visit during which a Pap smear was performed. Future reassessment will determine if these interventions increase follow-up care and, more importantly, the effect of these interventions upon the occurrence of more advanced cervical cancer.

## Supporting Information

S1 File Spanish Questionnaire. (PDF)

S2 File English Questionnaire. (PDF)
