# Supplementary material for: Barriers to Follow-Up for Abnormal Papanicolaou Smears among Female Sex Workers in Lima, Peru
Source: PLoS One. 2017 Jan 6;12(1):e0169327. doi: 10.1371/journal.pone.0169327 (PMC5217960; doi:10.1371/journal.pone.0169327)
Supplement: S1 File — (PDF) [file pone.0169327.s001.pdf]

Seguimiento al VPH entre trabajadoras sexuales en el Perú – Cuestionario

1. Número de identificación ________________________

Sección I – Información Demográfica

2. Año de Nacimiento ______

3. Lugar de Nacimiento _____________; ____________
(Pueblo/ciudad; país)

4. Educación
[]$_0$ Iletrado
[]$_1$ Letrado

5. Nivel de educación completado
[]$_1$ Título Profesional
[]$_2$ Título Universitario
[]$_3$ Escuela Secundaria
[]$_4$Parte de Escuela Secundaria
[]$_5$Escuela Primaria
[] Parte de Escuela Primaria

6. Total de años como trabajadora sexual ______________ Años

7. Estado de registro []$_1$ Registrada []$_0$ No registrada

8. Ingreso Mensual (soles)
[]$_1$<1000
[]$_2$ 1,001-2,000
[]$_3$ 2,001-5,000
[]$_4$ 5,001-10,000
[]$_5$>10,000
[]$_{-9}$No sabe

9. Estado Civil
[]$_1$ Soltera
[]$_2$ Casada
[]$_3$ Conviviente (no casada)
[]$_4$ Divorciada/separada
[]$_5$ Viuda

10. Edad de la primera relación sexual _______________ años

11. Número de embarazos _______________

12. Número de abortos _______________

13. Número de hijos vivos _______________

Sección II – Historia de análisis y tratamientos para infecciones de transmisión sexual:

14. ¿Alguna vez tuvo resultados positivos para alguna ETS? []$_1$ Si []$_0$No

Virus de Papiloma Humano

15. ¿Alguna vez dio positivo al virus del papiloma humano? **[]$_1$ S** **[]$_0$N Si no, pase al 16.**

15ª1. De ser Si, ¿Dónde se realizó el examen positivo?

[]$_1$ Centro de Salud Alberto Barton del Callao
[]$_2$ Otro lugar en Peru ___________
[]$_3$ Otro pais __________________

15ª2.¿Recibió tratamiento? **[]$_1$ S** **[]$_0$N Si no, pase al15h.**

De ser Sí:

15b. ¿Qué tratamiento recibió?

15b1. Medicina Oral	[]$_1$ S	[]$_0$ N
15b2. Medicina por inyección	[]$_1$ S	[]$_0$ N
15b3. Colposcopía	[]$_1$ S	[]$_0$ N
15b4. Cirugía	[]$_1$ S	[]$_0$ N
15b5. Consejería	[]$_1$ S	[]$_0$ N
15b6. No sabe	[]$_1$ S	[]$_0$ N

15c. ¿Dónde recibió el tratamiento?

15c1.Clínica Barton	[]$_1$ S	[]$_0$ N
15c2. Centro de derivación	[]$_1$ S	[]$_0$ N
15c3. Hospital Local	[]$_1$ S	[]$_0$ N
15c4. Otro__________________	[]$_1$ S	[]$_0$ N

15d. ¿Por qué fue a ese lugar?

15d1. Clínica de derivación	[]$_1$ S	[]$_0$ N
15d2. Ubicación conveniente	[]$_1$ S	[]$_0$ N
15d3.Amigo lo recomendó	[]$_1$ S	[]$_0$ N
15d4. Otro__________________	[]$_1$ S	[]$_0$ N

15e. ¿Cuánto tiempo pasó entre la fecha de su chequeo y la fecha de su primera cita para el tratamiento?

[]$_1$<2 semanas
[]$_2$ 2 semanas-1 mes
[]$_3$ 1-3 meses
[]$_4$ 3-6 meses
[]$_5$ 6 meses-1 año
[]$_6$ >1 año
[]$_{-9}$ No sabe

15f. ¿Fue informada de sus resultados? []$_1$ S []$_0$ N

15f1.De ser Si, ¿Qué le dijeron?

____

15g. ¿Se le pidió que regrese para realizar un seguimiento? []$_1$ S []$_0$N

15g1.De ser así, ¿Cuál es la frecuencia del seguimiento? _________

Si no recibió tratamiento:

15h. ¿Por qué no recibió un seguimiento?

15h1. No sabía a donde ir	[]$_1$ S	[]$_0$ N
15h2. Era difícil llegar al lugar	[]$_1$ S	[]$_0$ N
15h3. El costo era muy alto	[]$_1$ S	[]$_0$ N
15h4. No pensó que el tratamiento era importante	[]$_1$ S	[]$_0$ N
15h5. No tenía tiempo	[]$_1$ S	[]$_0$ N
15h6. Ansiedad/ Miedo	[]$_1$ S	[]$_0$ N
15h7. Otro (especificar) _________________	[]$_1$ S	[]$_0$ N

Cervicitis

16. ¿Alguna vez dio positivo para cervicitis? **[]$_1$ S** **[]$_0$N Si no, pase a la 17**

16ª1. De ser Si, ¿Dónde se realizó el examen positivo?

[]$_1$ Centro de Salud Alberto Barton del Callao

[]$_2$ Otro lugar en Peru ___________

[]$_3$ Otro pais __________________

16ª2. ¿Recibió tratamiento? **[]$_1$ S** **[]$_0$N Si no, pase a la 16h**.

De ser Sí:

16b. ¿Qué tratamiento recibió?

16b1. Medicina Oral	[]$_1$ S	[]$_0$ N
16b2. Medicina por inyección	[]$_1$ S	[]$_0$ N
16b3. Colposcopía	[]$_1$ S	[]$_0$ N
16b4. Cirugía	[]$_1$ S	[]$_0$ N
16b5. Consejería	[]$_1$ S	[]$_0$ N
16b6. No sabe	[]$_1$ S	[]$_0$ N

16c. ¿Dónde recibió el tratamiento?

16c1.Clínica Barton	[]$_1$ S	[]$_0$ N
16c2. Centro de derivación	[]$_1$ S	[]$_0$ N
16c3. Hospital Local	[]$_1$ S	[]$_0$ N
16c4. Otro__________________	[]$_1$ S	[]$_0$ N

16d. ¿Por qué fue a ese lugar?

16d1. Clínica de derivación	[]$_1$ S	[]$_0$ N
16d2. Ubicación conveniente	[]$_1$ S	[]$_0$ N
16d3.Amigo lo recomendó	[]$_1$ S	[]$_0$ N
16d4. Otro__________________	[]$_1$ S	[]$_0$ N

16e. ¿Cuánto tiempo pasó entre la fecha de su chequeo y la fecha de su primera cita para el tratamiento?

[]$_1$<2 semanas

[]$_2$ 2 semanas-1 mes

[]$_3$ 1-3 meses

[]$_4$ 3-6 meses

[]$_5$ 6 meses-1 año

[]$_6$ >1 año

[]$_{-9}$ No sabe

16f. ¿Fue informada de sus resultados? []$_1$ S []$_0$ N

16f1.De ser Si, ¿Qué le dijeron?

____

16g. ¿Se le pidió que regrese para realizar un seguimiento? []$_1$ S []$_0$N

16g1.De ser así, ¿Cuál es la frecuencia del seguimiento? _________

Si no recibió tratamiento:

16h. ¿Por qué no recibió un seguimiento?

16h1. No sabía a donde ir	[]$_1$ S	[]$_0$ N
16h2. Era difícil llegar al lugar	[]$_1$ S	[]$_0$ N
16h3. El costo era muy alto	[]$_1$ S	[]$_0$ N
16h4. No pensó que el tratamiento era importante	[]$_1$ S	[]$_0$ N
16h5. No tenía tiempo	[]$_1$ S	[]$_0$ N
16h6. Ansiedad/ Miedo	[]$_1$ S	[]$_0$ N
16h7. Otro (especificar) _________________	[]$_1$ S	[]$_0$ N

Sífilis

17. ¿Alguna vez dio positivo para Sífilis? []$_1$ S []$_0$N Si no, pase a la 18

17ª1. De ser Si, ¿Dónde se realizó el examen positivo?

[]$_1$ Centro de Salud Alberto Barton del Callao

[]$_2$ Otro lugar en Peru ___________

[]$_3$ Otro pais __________________

17ª2. ¿Recibió tratamiento? **[]$_1$ S []$_0$N Si no, pase a la 17h.**

De ser Sí:

17b. ¿Qué tratamiento recibió?

17b1. Medicina Oral []$_1$ S []$_0$ N

17b2. Medicina por inyección []$_1$ S []$_0$ N

17b3. Colposcopía []$_1$ S []$_0$ N

17b4. Cirugía []$_1$ S []$_0$ N

17b5. Consejería []$_1$ S []$_0$ N

17b6. No sabe []$_1$ S []$_0$ N

17c. ¿Dónde recibió el tratamiento?

17c1.Clínica Barton []$_1$ S []$_0$ N

17c2. Centro de derivación []$_1$ S []$_0$ N

17c3. Hospital Local []$_1$ S []$_0$ N

17c4. Otro__________________ []$_1$ S []$_0$ N

17d. ¿Por qué fue a ese lugar?

17d1. Clínica de derivación []$_1$ S []$_0$ N

17d2. Ubicación conveniente []$_1$ S []$_0$ N

17d3.Amigo lo recomendó []$_1$ S []$_0$ N

17d4. Otro__________________ []$_1$ S []$_0$ N

17e. ¿Cuánto tiempo pasó entre la fecha de su chequeo y la fecha de su primera cita para el tratamiento?

[]$_1$<2 semanas

[]$_2$ 2 semanas-1 mes

[]$_3$ 1-3 meses

[]$_4$ 3-6 meses

[]$_5$ 6 meses-1 año

[]$_6$ >1 año

[]$_{-9}$ No sabe

17f. ¿Fue informada de sus resultados? []$_1$ S []$_0$ N

17f1.De ser Si, ¿Qué le dijeron?

____

17g. ¿Se le pidió que regrese para realizar un seguimiento? []$_1$ S []$_0$N

17g1.De ser así, ¿Cuál es la frecuencia del seguimiento? ________

Si no recibió tratamiento:

17h. ¿Por qué no recibió un seguimiento?

17h1. No sabía a donde ir []$_1$ S []$_0$ N

17h2. Era difícil llegar al lugar []$_1$ S []$_0$ N

17h3. El costo era muy alto []$_1$ S []$_0$ N

17h4. No pensó que el tratamiento era importante []$_1$ S []$_0$ N

17h5. No tenía tiempo []$_1$ S []$_0$ N

17h6. Ansiedad/ Miedo []$_1$ S []$_0$ N

17h7. Otro (especificar) _________________ []$_1$ S []$_0$ N

Vaginosis Bacteriana

18. ¿Alguna vez dio positivo para Vaginosis Bacteriana? []$_1$ S []$_0$N Si no, pase a la 19

18ª1. De ser Si, ¿Dónde se realizó el examen positivo?

[]$_1$ Centro de Salud Alberto Barton del Callao

[]$_2$ Otro lugar en Peru ___________

[]$_3$ Otro pais __________________

18ª2. ¿Recibió tratamiento? **[]$_1$ S []$_0$N Si no, pase a la 18h.**

De ser Sí:

18b. ¿Qué tratamiento recibió?

18b1. Medicina Oral	[]$_1$ S	[]$_0$ N
18b2. Medicina por inyección	[]$_1$ S	[]$_0$ N
18b3. Colposcopía	[]$_1$ S	[]$_0$ N
18b4. Cirugía	[]$_1$ S	[]$_0$ N
18b5. Consejería	[]$_1$ S	[]$_0$ N
18b6. No sabe	[]$_1$ S	[]$_0$ N

18c. ¿Dónde recibió el tratamiento?

18c1.Clínica Barton	[]$_1$ S	[]$_0$ N
18c2. Centro de derivación	[]$_1$ S	[]$_0$ N
18c3. Hospital Local	[]$_1$ S	[]$_0$ N
18c4. Otro__________________	[]$_1$ S	[]$_0$ N

18d. ¿Por qué fue a ese lugar?

18d1. Clínica de derivación	[]$_1$ S	[]$_0$ N
18d2. Ubicación conveniente	[]$_1$ S	[]$_0$ N
18d3.Amigo lo recomendó	[]$_1$ S	[]$_0$ N
18d4. Otro__________________	[]$_1$ S	[]$_0$ N

18e. ¿Cuánto tiempo pasó entre la fecha de su chequeo y la fecha de su primera cita para el tratamiento?

[]$_1$<2 semanas

[]$_2$ 2 semanas-1 mes

[]$_3$ 1-3 meses

[]$_4$ 3-6 meses

[]$_5$ 6 meses-1 año

[]$_6$ >1 año

[]$_{-9}$ No sabe

18f. ¿Fue informada de sus resultados? []$_1$ S []$_0$ N

18f1.De ser Si, ¿Qué le dijeron?

____

18g. ¿Se le pidió que regrese para realizar un seguimiento? []$_1$ S []$_0$N

18g1.De ser así, ¿Cuál es la frecuencia del seguimiento? _________

Si no recibió tratamiento:

18h. ¿Por qué no recibió un seguimiento?

18h1. No sabía a donde ir	[]$_1$ S	[]$_0$ N
18h2. Era difícil llegar al lugar	[]$_1$ S	[]$_0$ N
18h3. El costo era muy alto	[]$_1$ S	[]$_0$ N
18h4. No pensó que el tratamiento era importante	[]$_1$ S	[]$_0$ N
18h5. No tenía tiempo	[]$_1$ S	[]$_0$ N
18h6. Ansiedad/ Miedo	[]$_1$ S	[]$_0$ N
18h7. Otro (especificar) _________________	[]$_1$ S	[]$_0$ N

Tricomoniasis

19. ¿Alguna vez dio positivo para Tricomoniasis? []$_1$S []$_0$N Si no, pase a la 20

19ª1. De ser Si, ¿Dónde se realizó el examen positivo?

[]$_1$ Centro de Salud Alberto Barton del Callao

[]$_2$ Otro lugar en Peru ___________

[]$_3$ Otro pais __________________

19ª2. ¿Recibió tratamiento? **[]$_1$S []$_0$N Si no, pase a la 19h.**

De ser Sí:

19b. ¿Qué tratamiento recibió?

19b1. Medicina Oral	[]$_1$ S	[]$_0$ N
19b2. Medicina por inyección	[]$_1$ S	[]$_0$ N
19b3. Colposcopía	[]$_1$ S	[]$_0$ N
19b4. Cirugía	[]$_1$ S	[]$_0$ N
19b5. Consejería	[]$_1$ S	[]$_0$ N
19b6. No sabe	[]$_1$ S	[]$_0$ N

19c. ¿Dónde recibió el tratamiento?

19c1.Clínica Barton	[]$_1$ S	[]$_0$ N
19c2. Centro de derivación	[]$_1$ S	[]$_0$ N
19c3. Hospital Local	[]$_1$ S	[]$_0$ N
19c4. Otro__________________	[]$_1$ S	[]$_0$ N

19d. ¿Por qué fue a ese lugar?

19d1. Clínica de derivación	[]$_1$ S	[]$_0$ N
19d2. Ubicación conveniente	[]$_1$ S	[]$_0$ N
19d3.Amigo lo recomendó	[]$_1$ S	[]$_0$ N
19d4. Otro__________________	[]$_1$ S	[]$_0$ N

19e. ¿Cuánto tiempo pasó entre la fecha de su chequeo y la fecha de su primera cita para el tratamiento?

[]$_1$<2 semanas

[]$_2$ 2 semanas-1 mes

[]$_3$ 1-3 meses

[]$_4$ 3-6 meses

[]$_5$ 6 meses-1 año

[]$_6$ >1 año

[]$_{-9}$ No sabe

19f. ¿Fue informada de sus resultados? []$_1$ S []$_0$ N

19f1.De ser Si, ¿Qué le dijeron?

____

19g. ¿Se le pidió que regrese para realizar un seguimiento? []$_1$ S []$_0$N

19g1.De ser así, ¿Cuál es la frecuencia del seguimiento? _________

Si no recibió tratamiento:

19h. ¿Por qué no recibió un seguimiento?

19h1. No sabía a donde ir	[]$_1$ S	[]$_0$ N
19h2. Era difícil llegar al lugar	[]$_1$ S	[]$_0$ N
19h3. El costo era muy alto	[]$_1$ S	[]$_0$ N
19h4. No pensó que el tratamiento era importante	[]$_1$ S	[]$_0$ N
19h5. No tenía tiempo	[]$_1$ S	[]$_0$ N
19h6. Ansiedad/ Miedo	[]$_1$ S	[]$_0$ N
19h7. Otro (especificar) _________________	[]$_1$ S	[]$_0$ N

Candida/Hongos

20. ¿Alguna vez dio positivo para Candida/Hongos? **[]$_1$S** **[]$_0$N Si no, pase a la 21**

20ª1. De ser Si, ¿Dónde se realizó el examen positivo?

[]$_1$ Centro de Salud Alberto Barton del Callao
[]$_2$ Otro lugar en Peru ___________
[]$_3$ Otro pais __________________

20ª2. ¿Recibió tratamiento? **[]$_1$S** **[]$_0$N Si no, pase a la 20h.**

De ser Sí:

20b. ¿Qué tratamiento recibió?

20b1. Medicina Oral []$_1$ S []$_0$ N
20b2. Medicina por inyección []$_1$ S []$_0$ N
20b3. Colposcopía []$_1$ S []$_0$ N
20b4. Cirugía []$_1$ S []$_0$ N
20b5. Consejería []$_1$ S []$_0$ N
20b6. No sabe []$_1$ S []$_0$ N

20c. ¿Dónde recibió el tratamiento?

20c1.Clínica Barton []$_1$ S []$_0$ N
20c2. Centro de derivación []$_1$ S []$_0$ N
20c3. Hospital Local []$_1$ S []$_0$ N
20c4. Otro__________________ []$_1$ S []$_0$ N

20d. ¿Por qué fue a ese lugar?

20d1. Clínica de derivación []$_1$ S []$_0$ N
20d2. Ubicación conveniente []$_1$ S []$_0$ N
20d3.Amigo lo recomendó []$_1$ S []$_0$ N
20d4. Otro__________________ []$_1$ S []$_0$ N

20e. ¿Cuánto tiempo pasó entre la fecha de su chequeo y la fecha de su primera cita para el tratamiento?

[]$_1$<2 semanas
[]$_2$ 2 semanas-1 mes
[]$_3$ 1-3 meses
[]$_4$ 3-6 meses
[]$_5$ 6 meses-1 año
[]$_6$ >1 año
[]$_{-9}$ No sabe

20f. ¿Fue informada de sus resultados? []$_1$ S []$_0$ N

20f1.De ser Si, ¿Qué le dijeron?

____

20g. ¿Se le pidió que regrese para realizar un seguimiento? []$_1$ S []$_0$N

20g1.De ser así, ¿Cuál es la frecuencia del seguimiento? _________

Si no recibió tratamiento:

20h. ¿Por qué no recibió un seguimiento?

20h1. No sabía a donde ir []$_1$ S []$_0$ N
20h2. Era difícil llegar al lugar []$_1$ S []$_0$ N
20h3. El costo era muy alto []$_1$ S []$_0$ N
20h4. No pensó que el tratamiento era importante []$_1$ S []$_0$ N
20h5. No tenía tiempo []$_1$ S []$_0$ N
20h6. Ansiedad/ Miedo []$_1$ S []$_0$ N
20h7. Otro (especificar) _________________ []$_1$ S []$_0$ N

Herpes Genital

21. ¿Alguna vez dio positivo para VIH? **[]$_1$S** **[]$_0$N Si no, pase a la 22**

21ª1. De ser Si, ¿Dónde se realizó el examen positivo?

[]$_1$ Centro de Salud Alberto Barton del Callao

[]$_2$ Otro lugar en Peru ___________

[]$_3$ Otro pais __________________

21ª2. ¿Recibió tratamiento? **[]$_1$S** **[]$_0$N Si no, pase a la 21h**.

De ser Sí:

21b. ¿Qué tratamiento recibió?

21b1. Medicina Oral	[]$_1$ S	[]$_0$ N
21b2. Medicina por inyección	[]$_1$ S	[]$_0$ N
21b3. Colposcopía	[]$_1$ S	[]$_0$ N
21b4. Cirugía	[]$_1$ S	[]$_0$ N
21b5. Consejería	[]$_1$ S	[]$_0$ N
21b6. No sabe	[]$_1$ S	[]$_0$ N

21c. ¿Dónde recibió el tratamiento?

21c1.Clínica Barton	[]$_1$ S	[]$_0$ N
21c2. Centro de derivación	[]$_1$ S	[]$_0$ N
21c3. Hospital Local	[]$_1$ S	[]$_0$ N
21c4. Otro__________________	[]$_1$ S	[]$_0$ N

21d. ¿Por qué fue a ese lugar?

21d1. Clínica de derivación	[]$_1$ S	[]$_0$ N
21d2. Ubicación conveniente	[]$_1$ S	[]$_0$ N
21d3.Amigo lo recomendó	[]$_1$ S	[]$_0$ N
21d4. Otro__________________	[]$_1$ S	[]$_0$ N

21e. ¿Cuánto tiempo pasó entre la fecha de su chequeo y la fecha de su primera cita para el tratamiento?

[]$_1$<2 semanas

[]$_2$ 2 semanas-1 mes

[]$_3$ 1-3 meses

[]$_4$ 3-6 meses

[]$_5$ 6 meses-1 año

[]$_6$ >1 año

[]$_{-9}$ No sabe

21f. ¿Fue informada de sus resultados? []$_1$ S []$_0$ N

21f1.De ser Si, ¿Qué le dijeron?

____

21g. ¿Se le pidió que regrese para realizar un seguimiento? []$_1$ S []$_0$N

21g1.De ser así, ¿Cuál es la frecuencia del seguimiento? _________

Si no recibió tratamiento:

21h. ¿Por qué no recibió un seguimiento?

21h1. No sabía a donde ir	[]$_1$ S	[]$_0$ N
21h2. Era difícil llegar al lugar	[]$_1$ S	[]$_0$ N
21h3. El costo era muy alto	[]$_1$ S	[]$_0$ N
21h4. No pensó que el tratamiento era importante	[]$_1$ S	[]$_0$ N
21h5. No tenía tiempo	[]$_1$ S	[]$_0$ N
21h6. Ansiedad/ Miedo	[]$_1$ S	[]$_0$ N
21h7. Otro (especificar) _________________	[]$_1$ S	[]$_0$ N

VIH

22. ¿Alguna vez dio positivo para VIH? []$_1$S []$_0$N Si no, pase a la 23

22ª1. De ser Si, ¿Dónde se realizó el examen positivo?

[]$_1$ Centro de Salud Alberto Barton del Callao

[]$_2$ Otro lugar en Peru ___________

[]$_3$ Otro pais __________________

22ª2. ¿Recibió tratamiento? **[]$_1$S []$_0$N Si no, pase a la 22h.**

De ser Sí:

22b. ¿Qué tratamiento recibió?

22b1. Medicina Oral []$_1$ S []$_0$ N

22b2. Medicina por inyección []$_1$ S []$_0$ N

22b3. Colposcopía []$_1$ S []$_0$ N

22b4. Cirugía []$_1$ S []$_0$ N

22b5. Consejería []$_1$ S []$_0$ N

22b6. No sabe []$_1$ S []$_0$ N

22c. ¿Dónde recibió el tratamiento?

22c1.Clínica Barton []$_1$ S []$_0$ N

22c2. Centro de derivación []$_1$ S []$_0$ N

22c3. Hospital Local []$_1$ S []$_0$ N

22c4. Otro__________________ []$_1$ S []$_0$ N

22d. ¿Por qué fue a ese lugar?

22d1. Clínica de derivación []$_1$ S []$_0$ N

22d2. Ubicación conveniente []$_1$ S []$_0$ N

22d3.Amigo lo recomendó []$_1$ S []$_0$ N

22d4. Otro__________________ []$_1$ S []$_0$ N

22e. ¿Cuánto tiempo pasó entre la fecha de su chequeo y la fecha de su primera cita para el tratamiento?

[]$_1$<2 semanas

[]$_2$ 2 semanas-1 mes

[]$_3$ 1-3 meses

[]$_4$ 3-6 meses

[]$_5$ 6 meses-1 año

[]$_6$ >1 año

[]$_{-9}$ No sabe

22f. ¿Fue informada de sus resultados? []$_1$ S []$_0$ N

22f1.De ser Si, ¿Qué le dijeron?

____

22g. ¿Se le pidió que regrese para realizar un seguimiento? []$_1$ S []$_0$N

22g1.De ser así, ¿Cuál es la frecuencia del seguimiento? _________

Si no recibió tratamiento:

22h. ¿Por qué no recibió un seguimiento?

22h1. No sabía a donde ir []$_1$ S []$_0$ N

22h2. Era difícil llegar al lugar []$_1$ S []$_0$ N

22h3. El costo era muy alto []$_1$ S []$_0$ N

22h4. No pensó que el tratamiento era importante []$_1$ S []$_0$ N

22h5. No tenía tiempo []$_1$ S []$_0$ N

22h6. Ansiedad/ Miedo []$_1$ S []$_0$ N

22h7. Otro (especificar) _________________ []$_1$ S []$_0$ N

Otras ETS

23. ¿Alguna vez dio positivo para una ETS que no fue mencionada? []$_1$S []$_0$N Si no, pase a la IV

23ª1. De ser Si, ¿Dónde se realizó el examen positivo?

[]$_1$ Centro de Salud Alberto Barton del Callao

[]$_2$ Otro lugar en Peru ___________

[]$_3$ Otro pais __________________

23ª2. ¿Recibió tratamiento? **[]$_1$S []$_0$N Si no, pase a la 23h.**

De ser Sí:

23b. ¿Qué tratamiento recibió?

23b1. Medicina Oral	[]$_1$ S	[]$_0$ N
23b2. Medicina por inyección	[]$_1$ S	[]$_0$ N
23b3. Colposcopía	[]$_1$ S	[]$_0$ N
23b4. Cirugía	[]$_1$ S	[]$_0$ N
23b5. Consejería	[]$_1$ S	[]$_0$ N
23b6. No sabe	[]$_1$ S	[]$_0$ N

23c. ¿Dónde recibió el tratamiento?

23c1.Clínica Barton	[]$_1$ S	[]$_0$ N
23c2. Centro de derivación	[]$_1$ S	[]$_0$ N
23c3. Hospital Local	[]$_1$ S	[]$_0$ N
23c4. Otro__________________	[]$_1$ S	[]$_0$ N

23d. ¿Por qué fue a ese lugar?

23d1. Clínica de derivación	[]$_1$ S	[]$_0$ N
23d2. Ubicación conveniente	[]$_1$ S	[]$_0$ N
23d3.Amigo lo recomendó	[]$_1$ S	[]$_0$ N
23d4. Otro__________________	[]$_1$ S	[]$_0$ N

23e. ¿Cuánto tiempo pasó entre la fecha de su chequeo y la fecha de su primera cita para el tratamiento?

[]$_1$<2 semanas

[]$_2$ 2 semanas-1 mes

[]$_3$ 1-3 meses

[]$_4$ 3-6 meses

[]$_5$ 6 meses-1 año

[]$_6$ >1 año

[]$_{-9}$ No sabe

23f. ¿Fue informada de sus resultados? []$_1$ S []$_0$ N

22f1.De ser Si, ¿Qué le dijeron?

____

23g. ¿Se le pidió que regrese para realizar un seguimiento? []$_1$ S []$_0$N

23g1.De ser así, ¿Cuál es la frecuencia del seguimiento? _________

Si no recibió tratamiento:

23h. ¿Por qué no recibió un seguimiento?

23h1. No sabía a donde ir	[]$_1$ S	[]$_0$ N
23h2. Era difícil llegar al lugar	[]$_1$ S	[]$_0$ N
23h3. El costo era muy alto	[]$_1$ S	[]$_0$ N
23h4. No pensó que el tratamiento era importante	[]$_1$ S	[]$_0$ N
23h5. No tenía tiempo	[]$_1$ S	[]$_0$ N
23h6. Ansiedad/ Miedo	[]$_1$ S	[]$_0$ N
23h7. Otro (especificar) _________________	[]$_1$ S	[]$_0$ N

Sección IV – Factores emocionales y psicológicos

Si el paciente dio positive para VPH, por favor complete la siguiente sección:
Respecto a sus resultados positivos para VPH:

24. Es importante tener un seguimiento para mis resultado(s) positivos.

1	2	3	4	5
Desacuerdo		*Ni de acuerdo ni en desacuerdo*		*De acuerdo*

25. Estoy consciente de las posibles consecuencias del(los) diagnóstico(s) sobre mi salud.

1	2	3	4	5
Desacuerdo		*Ni de acuerdo ni en desacuerdo*		*De acuerdo*

26. Mi infección es tratable.

1	2	3	4	5
Desacuerdo		*Ni de acuerdo ni en desacuerdo*		*De acuerdo*

27. Me siento cómoda durante el examen médico.

1	2	3	4	5
Desacuerdo		*Ni de acuerdo ni en desacuerdo*		*De acuerdo*

28. Me siento cómoda con los médicos.

1	2	3	4	5
Desacuerdo		*Ni de acuerdo ni en desacuerdo*		*De acuerdo*

29. Me siento avergonzada con mi(s) diagnóstico(s).

1	2	3	4	5
Desacuerdo		*Ni de acuerdo ni en desacuerdo*		*De acuerdo*

30. Me siento avergonzada de decir a otras personas sobre mi(s) diagnóstico(s).

1	2	3	4	5
Desacuerdo		*Ni de acuerdo ni en desacuerdo*		*De acuerdo*

31. Temo que pueda pasar m(s)i diagnóstico(s) a otros.

1	2	3	4	5
Desacuerdo		*Ni de acuerdo ni en desacuerdo*		*De acuerdo*

32. Temo desarrollar consecuencias adversas de largo plazo (como cáncer, infertilidad) como resultado de mi (s) diagnóstico(s).

1	2	3	4	5
Desacuerdo		*Ni de acuerdo ni en desacuerdo*		*De acuerdo*

33. Mi médico (doctor/enfermera/trabajador social) respondieron mis preguntas sobre mis resultados positivos.

1	2	3	4	5
Desacuerdo		*Ni de acuerdo ni en desacuerdo*		*De acuerdo*

34. Mi médico (doctor/enfermera/trabajador social) me explicó cómo obtener tratamiento para mis resultados positivos.

1	2	3	4	5
Desacuerdo		*Ni de acuerdo ni en desacuerdo*		*De acuerdo*

Si el paciente dio positive para una ETS diferente al VPH, por favor complete la siguiente sección:
Respecto a sus resultados positivos para ETS's (otra diferente a VPH):

35. Es importante tener un seguimiento para mis resultados positivos.
1 2 3 4 5
Desacuerdo *Ni de acuerdo ni en desacuerdo* *De acuerdo*

36. Mi infección es tratable.
1 2 3 4 5
Desacuerdo *Ni de acuerdo ni en desacuerdo* *De acuerdo*

37. Estoy consciente de las posibles consecuencias del diagnóstico sobre mi salud.
1 2 3 4 5
Desacuerdo *Ni de acuerdo ni en desacuerdo* *De acuerdo*

38. Me siento cómoda durante el examen médico.
1 2 3 4 5
Desacuerdo *Ni de acuerdo ni en desacuerdo* *De acuerdo*

39. Me siento cómoda con los médicos.
1 2 3 4 5
Desacuerdo *Ni de acuerdo ni en desacuerdo* *De acuerdo*

40. Me siento avergonzada con mi diagnóstico.
1 2 3 4 5
Desacuerdo *Ni de acuerdo ni en desacuerdo* *De acuerdo*

41. Me siento avergonzada de decir a otras personas sobre mi diagnóstico.
1 2 3 4 5
Desacuerdo *Ni de acuerdo ni en desacuerdo* *De acuerdo*

42. Temo que pueda pasar mi diagnóstico a otros.
1 2 3 4 5
Desacuerdo *Ni de acuerdo ni en desacuerdo* *De acuerdo*

43. Temo desarrollar consecuencias adversas de largo plazo (como cáncer, infertilidad) como resultado de mi diagnóstico.
1 2 3 4 5
Desacuerdo *Ni de acuerdo ni en desacuerdo* *De acuerdo*

44. Mi médico (doctor/enfermera/trabajador social) respondieron mis preguntas sobre mis resultados positivos.
1 2 3 4 5
Desacuerdo *Ni de acuerdo ni en desacuerdo* *De acuerdo*

45. Mi médico (doctor/enfermera/trabajador social) me explicó cómo obtener tratamiento para mis resultados positivos.
1 2 3 4 5
Desacuerdo *Ni de acuerdo ni en desacuerdo* *De acuerdo*

Sección V – Preguntas abiertas

Si el paciente dio positive para VPH, por favor llene la siguiente sección:

46. Por favor, díganos qué es lo que entiende sobre el resultado positivo del VPHP que recibió y las posibles consecuencias para su salud.

47. Si continuó con el seguimiento del VPH:

Por favor, díganos por qué decidió seguir con el tratamiento.

48. Si no continuó con el seguimiento del VPH

Por favor, díganos que le impidió seguir con el tratamiento.

Si el paciente dio positive para otra ETS, por favor llene la siguiente sección:

49. Por favor, díganos que entiende sobre sus resultados positivos para una ETS (diferente al VPH) y las posibles consecuencias sobre su salud.

50. Si continuó con el seguimiento de la ETS

Por favor, díganos por qué decidió seguir con el tratamiento.

51. Si no continuó con el seguimiento de la ETS:

Por favor, díganos que le impidió seguir con el tratamiento.
