# Supplementary material for: Barriers to Follow-Up for Abnormal Papanicolaou Smears among Female Sex Workers in Lima, Peru
Source: PLoS One. 2017 Jan 6;12(1):e0169327. doi: 10.1371/journal.pone.0169327 (PMC5217960; doi:10.1371/journal.pone.0169327)
Supplement: S2 File — (PDF) [file pone.0169327.s002.pdf]

Follow-up of HPV among female sex workers in Peru – Questionnaire

1. ID number ______________________

Section I – Demographic information

2. Date of Birth ___/___/_____
(dd/mm/yyyy)

3. Place of Birth ____________; ___________
(town/city; country)

4. Literacy:
[]$_{0}$Illiterate,
[]$_{1}$Literate

5. Level of education completed
[]$_{1}$Professional degree
[]$_{2}$University degree
[]$_{3}$Secondary school
[]$_{4}$Primary school
[]$_{5}$Some primary school

6. Total years employed as a sex worker _____________ years

7. Registration status []$_{1}$Registered []$_{0}$Unregistered

8. Monthly income (soles)
[]$_{1}$<1000
[]$_{2}$1,001-2,000
[]$_{3}$2,001-5,000
[]$_{4}$5,001-10,000
[]$_{5}$>10,000
[]$_{-9}$Don’t know

9. Marital status
[]$_{1}$Single
[]$_{2}$Married
[]$_{3}$Cohabitating (unmarried)
[]$_{4}$Divorced/separated
[]$_{5}$Widowed

10. Age at first intercourse ______________ years

11. Number of pregnancies ______________

12. Number of living children ______________

Section II – History of testing and treatment for sexually transmitted infections:

13. Did you ever test positive for any STI? []$_{1}$Y []$_{0}$N

Human Papilloma Virus

14. Did you ever test positive for human papilloma virus? **[]$_1$Y** **[]$_0$N** If no, skip to 15.

14a. If yes, did you receive treatment for this? []$_1$Y []$_0$N If no, skip to 14h.

If yes:

14b. Which treatments did you receive?

14b1. Oral medication []$_1$Y []$_0$N
14b2. Injection medication []$_1$Y []$_0$N
14b3. Colposcopy []$_1$Y []$_0$N
14b4. Surgery []$_1$Y []$_0$N
14b5. Counseling []$_1$Y []$_0$N
14b6. Don't know []$_1$Y []$_0$N

14c. Where did you receive treatment?

14c1. Barton Clinic []$_1$Y []$_0$N
14c2. Referral center []$_1$Y []$_0$N
14c3. Local hospital []$_1$Y []$_0$N
14c4. Other________________ []$_1$Y []$_0$N

14d. Why did you go to that location?

14d1. Clinical referral []$_1$Y []$_0$N
14d2. Convenient location []$_1$Y []$_0$N
14d3. Friend recommended []$_1$Y []$_0$N
14d4. Other________________ []$_1$Y []$_0$N

14e. How much time passed between the date of your screening test and the date of your first appointment for treatment?

[]$_1$<2 weeks
[]$_2$2 weeks-1 month
[]$_3$1-3 months
[]$_4$3-6 months
[]$_5$6 months-1 year
[]$_6$>1 year
[]$_{-9}$Don't know

14f. Were you informed of the results of your treatment or follow-up testing? []$_1$Y []$_0$N

14f1. If yes, what were you told?

____

14g. Were you instructed to return for further follow up care? []$_1$Y []$_0$N

14g1. If so, what is the frequency of follow up? __________

If you did not receive treatment:

14h. Why didn't you receive follow up care?

14h1. Did not know where to go []$_1$Y []$_0$N
14h2. Location was difficult to access []$_1$Y []$_0$N
14h3. Cost was prohibitive []$_1$Y []$_0$N
14h4. Did not think treatment was important []$_1$Y []$_0$N
14h5. Did not have time []$_1$Y []$_0$N
14h6. Anxiety/fear []$_1$Y []$_0$N
14h7. Other (specify)___________________________ []$_1$Y []$_0$N

Gonorrhea

15. Did you ever test positive for gonorrhea? **[]1Y** **[]0N** If no, skip to 16.

15a. If yes, did you receive treatment for this? []1Y []0N If no, skip to 15h.

If yes:

15b. Which treatments did you receive?

15b1. Oral medication []1Y []0N
15b2. Injection medication []1Y []0N
15b3. Colposcopy []1Y []0N
15b4. Surgery []1Y []0N
15b5. Counseling []1Y []0N
15b6. Don't know []1Y []0N

15c. Where did you receive treatment?

15c1. Barton Clinic []1Y []0N
15c2. Referral center []1Y []0N
15c3. Local hospital []1Y []0N
15c4. Other________________ []1Y []0N

15d. Why did you go to that location?

15d1. Clinical referral []1Y []0N
15d2. Convenient location []1Y []0N
15d3. Friend recommended []1Y []0N
15d4. Other________________ []1Y []0N

15e. How much time passed between the date of your screening test and the date of your first appointment for treatment?

[]1<2 weeks
[]22 weeks-1 month
[]31-3 months
[]43-6 months
[]56 months-1 year
[]6>1 year
[]-9Don't know

15f. Were you informed of the results of your treatment or follow-up testing? []1Y []0N

15f1. If yes, what were you told?

____

15g. Were you instructed to return for further follow up care? []1Y []0N

15g1. If so, what is the frequency of follow up? __________

If you did not receive treatment:

15h. Why didn't you receive follow up care?

15h1. Did not know where to go []1Y []0N
15h2. Location was difficult to access []1Y []0N
15h3. Cost was prohibitive []1Y []0N
15h4. Did not think treatment was important []1Y []0N
15h5. Did not have time []1Y []0N
15h6. Anxiety/fear []1Y []0N
15h7. Other (specify)___________________________ []1Y []0N

Chlamydia

16. Did you ever test positive for chlamydia? **[]$_1$Y** **[]$_0$N** If no, skip to 17.

16a. If yes, did you receive treatment for this? []$_1$Y []$_0$N If no, skip to 16h.

If yes:

16b. Which treatments did you receive?

16b1. Oral medication []$_1$Y []$_0$N
16b2. Injection medication []$_1$Y []$_0$N
16b3. Colposcopy []$_1$Y []$_0$N
16b4. Surgery []$_1$Y []$_0$N
16b5. Counseling []$_1$Y []$_0$N
16b6. Don't know []$_1$Y []$_0$N

16c. Where did you receive treatment?

16c1. Barton Clinic []$_1$Y []$_0$N
16c2. Referral center []$_1$Y []$_0$N
16c3. Local hospital []$_1$Y []$_0$N
16c4. Other________________ []$_1$Y []$_0$N

16d. Why did you go to that location?

16d1. Clinical referral []$_1$Y []$_0$N
16d2. Convenient location []$_1$Y []$_0$N
16d3. Friend recommended []$_1$Y []$_0$N
16d4. Other________________ []$_1$Y []$_0$N

16e. How much time passed between the date of your screening test and the date of your first appointment for treatment?

[]$_1$<2 weeks
[]$_2$2 weeks-1 month
[]$_3$1-3 months
[]$_4$3-6 months
[]$_5$6 months-1 year
[]$_6$>1 year
[]$_{-9}$Don't know

16f. Were you informed of the results of your treatment or follow-up testing? []$_1$Y []$_0$N

16f1. If yes, what were you told?

____

16g. Were you instructed to return for further follow up care? []$_1$Y []$_0$N

16g1. If so, what is the frequency of follow up? __________

If you did not receive treatment:

16h. Why didn't you receive follow up care?

16h1. Did not know where to go []$_1$Y []$_0$N
16h2. Location was difficult to access []$_1$Y []$_0$N
16h3. Cost was prohibitive []$_1$Y []$_0$N
16h4. Did not think treatment was important []$_1$Y []$_0$N
16h5. Did not have time []$_1$Y []$_0$N
16h6. Anxiety/fear []$_1$Y []$_0$N
16h7. Other (specify)___________________________ []$_1$Y []$_0$N

Syphilis

17. Did you ever test positive for syphilis? **[]$_1$Y** **[]$_0$N** If no, skip to 18.

17a. If yes, did you receive treatment for this? []$_1$Y []$_0$N If no, skip to 17h.

If yes:

17b. Which treatments did you receive?

17b1. Oral medication	[]$_1$Y	[]$_0$N
17b2. Injection medication	[]$_1$Y	[]$_0$N
17b3. Colposcopy	[]$_1$Y	[]$_0$N
17b4. Surgery	[]$_1$Y	[]$_0$N
17b5. Counseling	[]$_1$Y	[]$_0$N
17b6. Don't know	[]$_1$Y	[]$_0$N

17c. Where did you receive treatment?

17c1. Barton Clinic	[]$_1$Y	[]$_0$N
17c2. Referral center	[]$_1$Y	[]$_0$N
17c3. Local hospital	[]$_1$Y	[]$_0$N
17c4. Other________________	[]$_1$Y	[]$_0$N

17d. Why did you go to that location?

17d1. Clinical referral	[]$_1$Y	[]$_0$N
17d2. Convenient location	[]$_1$Y	[]$_0$N
17d3. Friend recommended	[]$_1$Y	[]$_0$N
17d4. Other________________	[]$_1$Y	[]$_0$N

17e. How much time passed between the date of your screening test and the date of your first appointment for treatment?

- []$_1$<2 weeks
- []$_2$2 weeks-1 month
- []$_3$1-3 months
- []$_4$3-6 months
- []$_5$6 months-1 year
- []$_6$>1 year
- []$_{-9}$Don't know

17f. Were you informed of the results of your treatment or follow-up testing? []$_1$Y []$_0$N

17f1. If yes, what were you told?

____

17g. Were you instructed to return for further follow up care? []$_1$Y []$_0$N

17g1. If so, what is the frequency of follow up? __________

If you did not receive treatment:

17h. Why didn't you receive follow up care?

17h1. Did not know where to go	[]$_1$Y	[]$_0$N
17h2. Location was difficult to access	[]$_1$Y	[]$_0$N
17h3. Cost was prohibitive	[]$_1$Y	[]$_0$N
17h4. Did not think treatment was important	[]$_1$Y	[]$_0$N
17h5. Did not have time	[]$_1$Y	[]$_0$N
17h6. Anxiety/fear	[]$_1$Y	[]$_0$N
17h7. Other (specify)___________________________	[]$_1$Y	[]$_0$N

Bacterial vaginosis

18. Did you ever test positive for bacterial vaginosis? **[]$_1$Y** **[]$_0$N** If no, skip to 19.

18a. If yes, did you receive treatment for this? []$_1$Y []$_0$N If no, skip to 18h.

If yes:

18b. Which treatments did you receive?

- 18b1. Oral medication []$_1$Y []$_0$N
- 18b2. Injection medication []$_1$Y []$_0$N
- 18b3. Colposcopy []$_1$Y []$_0$N
- 18b4. Surgery []$_1$Y []$_0$N
- 18b5. Counseling []$_1$Y []$_0$N
- 18b6. Don't know []$_1$Y []$_0$N

18c. Where did you receive treatment?

- 18c1. Barton Clinic []$_1$Y []$_0$N
- 18c2. Referral center []$_1$Y []$_0$N
- 18c3. Local hospital []$_1$Y []$_0$N
- 18c4. Other________________ []$_1$Y []$_0$N

18d. Why did you go to that location?

- 18d1. Clinical referral []$_1$Y []$_0$N
- 18d2. Convenient location []$_1$Y []$_0$N
- 18d3. Friend recommended []$_1$Y []$_0$N
- 18d4. Other________________ []$_1$Y []$_0$N

18e. How much time passed between the date of your screening test and the date of your first appointment for treatment?

- []$_1$<2 weeks
- []$_2$2 weeks-1 month
- []$_3$1-3 months
- []$_4$3-6 months
- []$_5$6 months-1 year
- []$_6$>1 year
- []$_{-9}$Don't know

18f. Were you informed of the results of your treatment or follow-up testing? []$_1$Y []$_0$N

14f1. If yes, what were you told?

____

18g. Were you instructed to return for further follow up care? []$_1$Y []$_0$N

18g1. If so, what is the frequency of follow up? __________

If you did not receive treatment:

18h. Why didn't you receive follow up care?

- 18h1. Did not know where to go []$_1$Y []$_0$N
- 18h2. Location was difficult to access []$_1$Y []$_0$N
- 18h3. Cost was prohibitive []$_1$Y []$_0$N
- 18h4. Did not think treatment was important []$_1$Y []$_0$N
- 18h5. Did not have time []$_1$Y []$_0$N
- 18h6. Anxiety/fear []$_1$Y []$_0$N
- 18h7. Other (specify)___________________________ []$_1$Y []$_0$N

Trichomoniasis

19. Did you ever test positive for trichomoniasis? **[]$_1$Y** **[]$_0$N** If no, skip to 20.

19a. If yes, did you receive treatment for this? []$_1$Y []$_0$N If no, skip to 19h.

If yes:

19b. Which treatments did you receive?

- 19b1. Oral medication []$_1$Y []$_0$N
- 19b2. Injection medication []$_1$Y []$_0$N
- 19b3. Colposcopy []$_1$Y []$_0$N
- 19b4. Surgery []$_1$Y []$_0$N
- 19b5. Counseling []$_1$Y []$_0$N
- 19b6. Don't know []$_1$Y []$_0$N

19c. Where did you receive treatment?

- 19c1. Barton Clinic []$_1$Y []$_0$N
- 19c2. Referral center []$_1$Y []$_0$N
- 19c3. Local hospital []$_1$Y []$_0$N
- 19c4. Other________________ []$_1$Y []$_0$N

19d. Why did you go to that location?

- 19d1. Clinical referral []$_1$Y []$_0$N
- 19d2. Convenient location []$_1$Y []$_0$N
- 19d3. Friend recommended []$_1$Y []$_0$N
- 19d4. Other________________ []$_1$Y []$_0$N

19e. How much time passed between the date of your screening test and the date of your first appointment for treatment?

- []$_1$<2 weeks
- []$_2$2 weeks-1 month
- []$_3$1-3 months
- []$_4$3-6 months
- []$_5$6 months-1 year
- []$_6$>1 year
- []$_{-9}$Don't know

19f. Were you informed of the results of your treatment or follow-up testing? []$_1$Y []$_0$N

19f1. If yes, what were you told?

____

19g. Were you instructed to return for further follow up care? []$_1$Y []$_0$N

19g1. If so, what is the frequency of follow up? __________

If you did not receive treatment:

19h. Why didn't you receive follow up care?

- 19h1. Did not know where to go []$_1$Y []$_0$N
- 19h2. Location was difficult to access []$_1$Y []$_0$N
- 19h3. Cost was prohibitive []$_1$Y []$_0$N
- 19h4. Did not think treatment was important []$_1$Y []$_0$N
- 19h5. Did not have time []$_1$Y []$_0$N
- 19h6. Anxiety/fear []$_1$Y []$_0$N
- 19h7. Other (specify)___________________________ []$_1$Y []$_0$N

HIV

20. Did you ever test positive for HIV? **[]$_1$Y** **[]$_0$N** If no, skip to 21.

20a. If yes, did you receive treatment for this? []$_1$Y []$_0$N If no, skip to 20h.

If yes:

20b. Which treatments did you receive?

- 20b1. Oral medication []$_1$Y []$_0$N
- 20b2. Injection medication []$_1$Y []$_0$N
- 20b3. Colposcopy []$_1$Y []$_0$N
- 20b4. Surgery []$_1$Y []$_0$N
- 20b5. Counseling []$_1$Y []$_0$N
- 20b6. Don't know []$_1$Y []$_0$N

20c. Where did you receive treatment?

- 20c1. Barton Clinic []$_1$Y []$_0$N
- 20c2. Referral center []$_1$Y []$_0$N
- 20c3. Local hospital []$_1$Y []$_0$N
- 20c4. Other________________ []$_1$Y []$_0$N

20d. Why did you go to that location?

- 20d1. Clinical referral []$_1$Y []$_0$N
- 20d2. Convenient location []$_1$Y []$_0$N
- 20d3. Friend recommended []$_1$Y []$_0$N
- 20d4. Other________________ []$_1$Y []$_0$N

20e. How much time passed between the date of your screening test and the date of your first appointment for treatment?

- []$_1$<2 weeks
- []$_2$2 weeks-1 month
- []$_3$1-3 months
- []$_4$3-6 months
- []$_5$6 months-1 year
- []$_6$>1 year
- []$_{-9}$Don't know

20f. Were you informed of the results of your treatment or follow-up testing? []$_1$Y []$_0$N

20f1. If yes, what were you told?

____

20g. Were you instructed to return for further follow up care? []$_1$Y []$_0$N

20g1. If so, what is the frequency of follow up? __________

If you did not receive treatment:

20h. Why didn't you receive follow up care?

- 20h1. Did not know where to go []$_1$Y []$_0$N
- 20h2. Location was difficult to access []$_1$Y []$_0$N
- 20h3. Cost was prohibitive []$_1$Y []$_0$N
- 20h4. Did not think treatment was important []$_1$Y []$_0$N
- 20h5. Did not have time []$_1$Y []$_0$N
- 20h6. Anxiety/fear []$_1$Y []$_0$N
- 20h7. Other (specify)____________________________ []$_1$Y []$_0$N

Genital herpes

21. Did you ever test positive for genital herpes? **[]$_1$Y** **[]$_0$N** If no, skip to 22.

21a. If yes, did you receive treatment for this? []$_1$Y []$_0$N If no, skip to 21h.

If yes:

21b. Which treatments did you receive?

- 21b1. Oral medication []$_1$Y []$_0$N
- 21b2. Injection medication []$_1$Y []$_0$N
- 21b3. Colposcopy []$_1$Y []$_0$N
- 21b4. Surgery []$_1$Y []$_0$N
- 21b5. Counseling []$_1$Y []$_0$N
- 21b6. Don’t know []$_1$Y []$_0$N

21c. Where did you receive treatment?

- 21c1. Barton Clinic []$_1$Y []$_0$N
- 21c2. Referral center []$_1$Y []$_0$N
- 21c3. Local hospital []$_1$Y []$_0$N
- 21c4. Other________________ []$_1$Y []$_0$N

21d. Why did you go to that location?

- 21d1. Clinical referral []$_1$Y []$_0$N
- 21d2. Convenient location []$_1$Y []$_0$N
- 21d3. Friend recommended []$_1$Y []$_0$N
- 21d4. Other________________ []$_1$Y []$_0$N

21e. How much time passed between the date of your screening test and the date of your first appointment for treatment?

- []$_1$<2 weeks
- []$_2$2 weeks-1 month
- []$_3$1-3 months
- []$_4$3-6 months
- []$_5$6 months-1 year
- []$_6$>1 year
- []$_{-9}$Don’t know

21f. Were you informed of the results of your treatment or follow-up testing? []$_1$Y []$_0$N

21f1. If yes, what were you told?

____

21g. Were you instructed to return for further follow up care? []$_1$Y []$_0$N

21g1. If so, what is the frequency of follow up? __________

If you did not receive treatment:

21h. Why didn’t you receive follow up care?

- 21h1. Did not know where to go []$_1$Y []$_0$N
- 21h2. Location was difficult to access []$_1$Y []$_0$N
- 21h3. Cost was prohibitive []$_1$Y []$_0$N
- 21h4. Did not think treatment was important []$_1$Y []$_0$N
- 21h5. Did not have time []$_1$Y []$_0$N
- 21h6. Anxiety/fear []$_1$Y []$_0$N
- 21h7. Other (specify)__________________________ []$_1$Y []$_0$N

Other STIs

22. Did you ever test positive for another STI not mentioned? []$_1$Y []$_0$N If no, skip to Section IV.

22a. If yes, did you receive treatment for this? []$_1$Y []$_0$N If no, skip to 22h.

If yes:

22b. Which treatments did you receive?

- 22b1. Oral medication []$_1$Y []$_0$N
- 22b2. Injection medication []$_1$Y []$_0$N
- 22b3. Colposcopy []$_1$Y []$_0$N
- 22b4. Surgery []$_1$Y []$_0$N
- 22b5. Counseling []$_1$Y []$_0$N
- 22b6. Don't know []$_1$Y []$_0$N

22c. Where did you receive treatment?

- 22c1. Barton Clinic []$_1$Y []$_0$N
- 22c2. Referral center []$_1$Y []$_0$N
- 22c3. Local hospital []$_1$Y []$_0$N
- 22c4. Other________________ []$_1$Y []$_0$N

22d. Why did you go to that location?

- 22d1. Clinical referral []$_1$Y []$_0$N
- 22d2. Convenient location []$_1$Y []$_0$N
- 22d3. Friend recommended []$_1$Y []$_0$N
- 22d4. Other________________ []$_1$Y []$_0$N

22e. How much time passed between the date of your screening test and the date of your first appointment for treatment?

- []$_1$<2 weeks
- []$_2$2 weeks-1 month
- []$_3$1-3 months
- []$_4$3-6 months
- []$_5$6 months-1 year
- []$_6$>1 year
- []$_{-9}$Don't know

22f. Were you informed of the results of your treatment or follow-up testing? []$_1$Y []$_0$N

22f1. If yes, what were you told?

____

22g. Were you instructed to return for further follow up care? []$_1$Y []$_0$N

22g1. If so, what is the frequency of follow up? __________

If you did not receive treatment:

22h. Why didn't you receive follow up care?

- 22h1. Did not know where to go []$_1$Y []$_0$N
- 22h2. Location was difficult to access []$_1$Y []$_0$N
- 22h3. Cost was prohibitive []$_1$Y []$_0$N
- 22h4. Did not think treatment was important []$_1$Y []$_0$N
- 22h5. Did not have time []$_1$Y []$_0$N
- 22h6. Anxiety/fear []$_1$Y []$_0$N
- 22h7. Other (specify)___________________________ []$_1$Y []$_0$N

Section IV – Emotional and psychological factors

If the patient tested positive for HPV, please complete the following section:

Regarding your positive HPV test result:

23. It is important to obtain follow-up care for my positive test result.
1 2 3 4 5
Disagree *Neither agree or disagree* *Agree*

24. I understand the possible consequences of my diagnosis to my health.
1 2 3 4 5
Disagree *Neither agree or disagree* *Agree*

25. My infection is treatable.
1 2 3 4 5
Disagree *Neither agree or disagree* *Agree*

26. I feel comfortable during the medical exam.
1 2 3 4 5
Disagree *Neither agree or disagree* *Agree*

27. I feel comfortable with medical providers.
1 2 3 4 5
Disagree *Neither agree or disagree* *Agree*

28. I feel ashamed about my diagnosis.
1 2 3 4 5
Disagree *Neither agree or disagree* *Agree*

29. I am embarrassed to tell other people about my diagnosis.
1 2 3 4 5
Disagree *Neither agree or disagree* *Agree*

30. I fear that I may pass my diagnosis to others.
1 2 3 4 5
Disagree *Neither agree or disagree* *Agree*

31. I fear that I may develop long-term adverse consequences (e.g. cancer, infertility) as a result of my diagnosis.
1 2 3 4 5
Disagree *Neither agree or disagree* *Agree*

32. My clinician (doctor/nurse/social worker) answered my questions about my positive test result.
1 2 3 4 5
Disagree *Neither agree or disagree* *Agree*

33. My clinician (doctor/nurse/social worker) explained how to get treatment for my positive test result.
1 2 3 4 5
Disagree *Neither agree or disagree* *Agree*

If the patient tested positive for any STI other than HPV, please complete the following section:

Regarding your positive STI test result (other than HPV):

34. It is important to obtain follow-up care for my positive test result(s).
1 2 3 4 5
Disagree *Neither agree or disagree* *Agree*

35. My infection is treatable.
1 2 3 4 5
Disagree *Neither agree or disagree* *Agree*

36. I understand the possible consequences of my diagnosis(es) to my health.
1 2 3 4 5
Disagree *Neither agree or disagree* *Agree*

37. I feel comfortable during the medical exam.
1 2 3 4 5
Disagree *Neither agree or disagree* *Agree*

38. I feel comfortable with medical providers.
1 2 3 4 5
Disagree *Neither agree or disagree* *Agree*

39. I feel ashamed about my diagnosis(es).
1 2 3 4 5
Disagree *Neither agree or disagree* *Agree*

40. I am embarrassed to tell other people about my diagnosis(es).
1 2 3 4 5
Disagree *Neither agree or disagree* *Agree*

41. I fear that I may pass my diagnosis(es) to others.
1 2 3 4 5
Disagree *Neither agree or disagree* *Agree*

42. I fear that I may develop long-term adverse consequences (e.g. cancer, infertility) as a result of my diagnosis(es).
1 2 3 4 5
Disagree *Neither agree or disagree* *Agree*

43. My clinician (doctor/nurse/social worker) answered my questions about my positive test result.
1 2 3 4 5
Disagree *Neither agree or disagree* *Agree*

44. My clinician (doctor/nurse/social worker) explained how to get treatment for my positive test result.
1 2 3 4 5
Disagree *Neither agree or disagree* *Agree*

Section V – Open-ended questions

If the patient tested positive for HPV, please complete the following section:

45. Please tell us what you understand about the positive HPV test result you received, and the possible consequences to your health.

46. If you pursued follow up care for HPV:

Please tell us why you decided to pursue treatment.

47. If you did not pursue follow up care HPV:

Please tell us what prevented you from pursuing treatment.

If the patient tested positive for any other STI, please complete the following section:

48. Please tell us what you understand about the positive STI test result (other than HPV) you received, and the possible consequences to your health.

49. If you pursued follow up care for other STIs:

Please tell us why you decided to pursue treatment.

50. If you did not pursue follow up care for other STIs:

Please tell us what prevented you from pursuing treatment.
